# Measuring the aggregated impact of research: Establishing criteria for coding Translational Science Benefits Model data

**DOI:** 10.1017/cts.2025.76

**Published:** 2025-05-16

**Authors:** Nicole Miovsky, Amanda Woodworth, Stephanie Andersen, Rosalina Das, Julie Heidbreder, Rechelle Paranal, Clara M. Pelfrey, Jessica Sperling, Beth Tigges, Boris B. Volkov, Margaret Schneider

**Affiliations:** 1 Institute for Clinical and Translational Science, University of California Irvine, Irvine, CA, USA; 2 Clark-Fox Policy Institute, Brown School at Washington University in St. Louis, St Louis, MO, USA; 3 Miami Clinical and Translational Science Institute, University of Miami, Miami, FL, USA; 4 Center for Public Health Systems Science, Brown School at Washington University in St. Louis, St. Louis, MO, USA; 5 South Carolina Clinical and Translational Research Institute, Medical University of South Carolina, Charleston, SC, USA; 6 Clinical and Translational Science Collaborative of Northern Ohio, Case Western Reserve University, Cleveland, OH, USA; 7 Clinical and Translational Science Institute, Duke University School of Medicine, Durham, NC, USA; 8 Social Science Research Institute, Duke University, Durham, NC, USA; 9 University of New Mexico Health Sciences Clinical and Translational Science Center, Albuquerque, NM, USA; 10 University of Minnesota Clinical and Translational Science Institute, Institute for Health Informatics, Minneapolis, MN, USA

**Keywords:** Research impact evaluation, societal impact, health research, coding criteria, content validation, Delphi panel

## Abstract

**Introduction:**

A promising approach to assessing research impact draws on the Translational Science Benefits Model (TSBM), an evaluation model that tracks the applied benefits of research in four domains: Clinical and Medical; Community and Public Health; Economic; and Policy and Legislative. However, standardized methods to verify TSBM benefit data, to aid in aggregating impact data within quantitative summaries, do not currently exist.

**Methods:**

A panel of 11 topic experts participated in a modified Delphi process for establishing content and face validity of a set of criteria for verifying qualitative TSBM data. Two survey rounds were completed by panelists, with a moderated discussion in between rounds to discuss criteria not reaching consensus. Criteria with panel consensus at or above 70% in the survey rounds were confirmed as validated.

**Results:**

Criteria fell into 9 categories: Content Relevant, Project Related, Who, Reach, What, How, Novel, Documented Evidence, and When. The Delphi process yielded 197 total criteria across the 30 benefits characterized by the TSBM (range = 5–8 criteria per benefit).

**Discussion:**

The results of this Delphi process lay the foundation for developing a TSBM coding tool for evaluating and quantifying TSBM data. Standardizing this process will enable data aggregation, group analysis, and the comparison of research impact across contexts.

## Introduction

Research impact, broadly defined as the creation of knowledge or tools to make positive changes in the real world [1], is inherently complex to measure. There are several challenges to tracking and evaluating impact; notably, the lengthy and cumulative nature of research impact [2]; the barriers to accessing information about outcomes that can occur years after typical project funding cycles have ended [3]; and the subjectivity inherent in the perceived benefits coming from research [4]. Adding further to the complexity is the current lack of high-quality, valid measures and tools to assess research impact [5]. However, it is important to evaluate, since impact bridges scientific advances to practical application outside of academia, and resources are increasingly being allocated according to perceived societal impact of research activities [6–8]. It is critically important to assess health impacts beyond academic output, especially given the historically inconsistent and protracted translation [9,10] of research findings into practical outcomes that benefit human health.

Academic metrics, such as publications and grants, have commonly been used as an indicator of research impact. Bibliometrics is a commonly used quantitative method; for example, using citation counts to measure publication impact [11]. This approach provides a useful measure of research productivity but does not assess whether research findings are being applied in the real world (e.g., health care settings). A more recent impact metric that is gaining popularity is alternative metrics (altmetrics), which are web-based metrics recording online engagement for a publication, which can include downloads, clicks, tweets, bookmarks, and saves [12]. Altmetrics suggest the visibility of a publication but still lack information on the impact research has within applied settings and for society at large. Other recent approaches assess research collaborations, such as Social Network Analysis, which assesses collaborative structures and the impact they have on the research enterprise [13,14]. Social Network Analysis can collect substantial information about interactions between researchers and external stakeholders that enable future impact, and may identify progress towards societal benefits [5], but does not specifically evaluate the resulting benefits.

In 2017, program evaluators at the Washington University in St Louis (referred to here as Washington University) presented a new model to measure the impact of research projects in applied settings: the Translational Science Benefits Model (TSBM) [15]. The TSBM is an evolving framework for assessing the societal impacts that result from research projects. Currently, it includes 30 potential research benefits across 4 domains: Clinical and Medical; Community and Public Health; Economic; and Policy and Legislative [15]. TSBM has been gaining traction within the research community, as suggested by almost 50 case studies published on the TSBM website [16] and the increase in publications referencing TSBM (from 5 before 2022 to more than 30 after). Resources developed by Washington University focus on data collection to create case studies and short impact vignettes that highlight the benefits emerging from a single project [17,18].

Case studies are an appealing format to highlight the impact of a specific project as they can provide a rich and complex picture of a single project [19] using data often obtained through interviews or diligent document review [20,21]. Case studies may also enable comparisons between small sets of studies using a qualitative or narrative approach. When the goal is to examine the aggregate impact of a group of studies, however, the case study format becomes more difficult to use. Many research enterprises are supporting multiple projects concurrently (comprising a “research portfolio”) and are required to periodically summarize the impact of their research portfolios for funders and other stakeholders. Case studies are useful as illustrations of impact, but they are a resource-intensive approach that is not easily scalable for describing impact across a large research portfolio or program. Surveys with forced-choice items (e.g., “yes” or “no”) are an alternative strategy to collecting TSBM data that are less resource-intensive and streamlines data collection. A mixed-methods approach that combines forced-choice items with narrative text has shown promise for collecting information on TSBM benefits in a manner that facilitates aggregation across multiple projects [22].

Two challenges are present when analyzing this type of qualitative TSBM data. First, it may be subject to misreporting by respondents owing to varying interpretations of what constitutes a specific translational science benefit [22]. Additionally, the process of coding the qualitative data may be subject to variability across coders operating with different implicit assumptions about what constitutes sufficient evidence that a research project has generated a given benefit. These challenges potentially introduce bias into the data. One solution is to standardize a set of coding criteria for determining, based on a narrative description of the research, whether a reported benefit can reasonably be said to have resulted from the project.

A systematic methodology for assessing qualitative TSBM data would be a valuable tool for producing both qualitative and quantitative summaries of the translational impacts of a research portfolio. Additionally, quantitative reporting enables the evaluation of initiatives over time and across sites and could lead to breakthroughs in research management that accelerate the translation of discoveries into applications. In this report, we describe the results of a Delphi panel process to determine the face and content validity of a proposed set of criteria to determine whether each of the TSBM benefits resulted from a given line of research.

## Methods

### Study context

The concept of standardizing a method for TSBM data analysis arose at the University of California, Irvine (UCI) Institute for Clinical and Translational Sciences (ICTS), and led to developing a set of potential TSBM coding criteria. ICTS has been a Clinical Translational Science Award (CTSA) site funded by the CTSA mechanism since 2010, and as required by the award, it has funded a cohort of translational pilot studies each year. Starting in 2020, ICTS began collecting TSBM information from pilot study investigators during their annual progress reporting. Using an online survey, funded investigators indicated which of the TSBM benefits had resulted from their projects in closed-ended items and were asked to support the assertion with a description of the benefit in an open-ended text box. For example, when a researcher reported that their project had generated a benefit within the Clinical and Medical domain of the TSBM, the researcher was asked which of the 9 specific Clinical or Medical benefits within the TSBM resulted from their work, and was prompted to describe this benefit in an open text box. During the process of assessing the qualitative data, the UCI team discovered that a substantial proportion of the self-reported benefits were unable to be confirmed as demonstrated using the investigator-provided text, which either described activities not relevant to the selected benefit, or lacked critical details needed to verify that the benefit had occurred [22].

The UCI team therefore developed a systematic process to evaluate the evidence provided by researchers for the TSBM benefits they reported. Coding categories were developed to explain the breadth of information needed to verify reported impacts, which were loosely based on the “journalistic six” (Who, What, When, Where, Why, and How). For each benefit, a set of criteria informed by these categories and tailored to the given benefit was created for coders to assess. For instance, extending the example above, if an investigator reported that a study led to a new diagnostic procedure, the proposed criteria for evaluating the qualitative information provided by the investigator would include: 1) content that is relevant to the TSBM definition of a diagnostic procedure; 2) content indicating the benefit is tied to the research project; 3) who the diagnostic procedure is for; 4) what the purpose of the diagnostic procedure is; 5) if it is new/ novel or has improved on previous diagnostic procedures; and 6) content indicating the benefit had already resulted from the research.

Informed by the evaluation of qualitative data for benefits (*n* = 79) reported in ICTS progress reports, the UCI team proposed criteria for all of the 30 TSBM benefits. The set of criteria that emerged from this coding experience formed the basis of the current study to establish face and content validity of these criteria, with the ultimate goal to standardize a process for characterizing the translational science benefits that have emerged from given research.

### Participants

A panel of TSBM experts was recruited using a screening survey administered through REDCap [23,24]. REDCap is a secure, web-based software platform designed to support data capture for research studies, providing 1) an intuitive interface with built-in checks for accurate data capture; 2) audit trails for tracking data manipulation and export procedures; 3) automated export procedures for seamless data downloads to common statistical packages; and 4) procedures for data integration and interoperability with external sources. The screening survey was sent to evaluators and staff who were members of the Association for Clinical and Translational Science Evaluation Special Interest Group or the CTSA Evaluators’ TSBM Working Group. Snowball sampling was employed to identify and screen additional contacts knowledgeable about TSBM. The screening survey asked about experience with collecting, analyzing, and reporting TSBM data. To be included, panelists were required to show multiple forms of experience with TSBM data collection and analysis for qualitative and/or quantitative methods.

### Procedures

#### Delphi panel

The Delphi methodology is a well-known approach for reaching consensus on an emerging topic and for generating guidelines or standards for which there is minimal research evidence [25]. The Delphi method utilizes a panel of experts who participate in repeated rounds of anonymous feedback to reach a consensus. Typical characteristics of the Delphi method include iteration, anonymity, and controlled feedback leading to group consensus. Described below are the steps completed for this Delphi Panel.

**Onboarding.** Panelists were invited to attend a one-hour orientation about the TSBM coding project. An onboarding packet was provided, which included the coding category descriptions, a list of the proposed coding criteria for each TSBM benefit, and the TSBM definitions and rationale as published by Washington University [26]. Panelists were required either to attend the orientation meeting on Zoom or watch a recording of the orientation.

**Round 1 of Delphi Panel.** Round 1 consisted of panelists completing an online Benefits Criteria Survey (details provided in Measures). The survey was administered using REDCap [23,24]. Criteria were considered validated (i.e., have face and content validity) if the panelists reached consensus with >70% panelist agreement. The criteria on which consensus was not reached were moved on for discussion and further panel consideration, as detailed below.

**Delphi Panel Moderated Discussion.** A modification of a typical Delphi method was used to include a step for panel discussion of the non-consensus criteria. Following the Round 1 survey, a summary of the results of the Round 1 Benefits Criteria Survey was shared with panelists and then discussed in a recorded 90-minute meeting on Zoom. The discussion was moderated by the first and second authors. A digital interactive whiteboard was used as a shared space for panelists to post online about the remaining criteria before, during, and for a day after the discussion.

**Round 2 of Delphi Panel.** Panelists who participated in the moderated discussion or viewed the recording were invited to join in a second round of the Benefits Criteria Survey to evaluate criteria that had not reached consensus or were newly suggested by panelists in Round 1. Criteria from Round 2 that reached the 70% consensus threshold were confirmed as validated (i.e., have face and content validity).

### Measures

#### Benefits criteria survey

The Benefits Criteria Survey (BCS) for Round 1 of the Delphi process included all proposed criteria for TSBM benefits, drawn from eight categories: Content Relevant, Project Related, Who, Reach, What, How, Novel, and When. Descriptions of the categories are provided in Table 1. Not all categories were deemed as relevant to every benefit, resulting in a tailored set of criteria across each of the 30 TSBM benefits, for a total number of 167 criteria proposed in the BCS Round 1 survey. Within the survey, panelists were asked to consider “In your view, are the criteria below required to demonstrate this benefit?.” Response options were “yes, required” or “no, not required.” Additionally, panelists could propose new criteria. There was also an open text field for respondents to write comments about their thought process on the set of criteria for each benefit.

**Table 1.** Explanations of the eight proposed categories

Categories	Explanation
**Content Relevant**	Response includes content with a connection to the benefit described in the TSBM definition (i.e., has face validity).
**Project Related**	Response indicates the benefit was related to or comes from project activities.
**Who**	Response explains “who,” such as the entity issuing and/or providing the benefit, or the individual/group who received the benefit (as applicable).
**Reach**	Response describes a reach beyond the research project, consistent with the size of reach in the benefit definition.
**What**	Response has sufficient details to describe the project activities or project outcomes related to the benefit.
**How**	Response has sufficient details to describe the way project activities were done related to the benefit.
**Novel**	Response explains how the project activity was new/novel or what improvement relevant to the benefit was made.
**When**	Response indicates the benefit has occurred and is not solely in the future.

In Round 2 of the BCS, panelists completed a condensed version of the survey containing only the criteria on which the panelists had not reached consensus in Round 1, as well as the new criteria suggested by panelists. The ability to suggest additional criteria was removed in Round 2. The open text field for respondent’s feedback on criteria was retained.

## Results

### Delphi panel participants

Of the 20 individuals who completed the screening survey, 12 (60%) were eligible based on experience with TSBM data collection and qualitative or quantitative analysis of TSBM data, and 11 (55%) agreed to join the panel. The panel was comprised of evaluators and staff affiliated with a CTSA hub from eight universities. Most panelists self-rated as having “a lot of experience = 5” with TSBM overall (on average *M* = 3.86 out of 5). The 11 panelists included three members of the TSBM group at Washington University, one of whom helped develop the initial TSBM framework and benefit definitions. The experience that panelists had overall with TSBM consisted of incorporating TSBM data into hub data collection (100% of panelists), qualitative or quantitative data analysis of TSBM data (100% of panelists), development of case studies (73% of panelists), and quantitative reporting and/or data visualization of TSBM data (91% of panelists).

### Benefits criteria survey round 1 and moderated discussion

**Figure 1.**
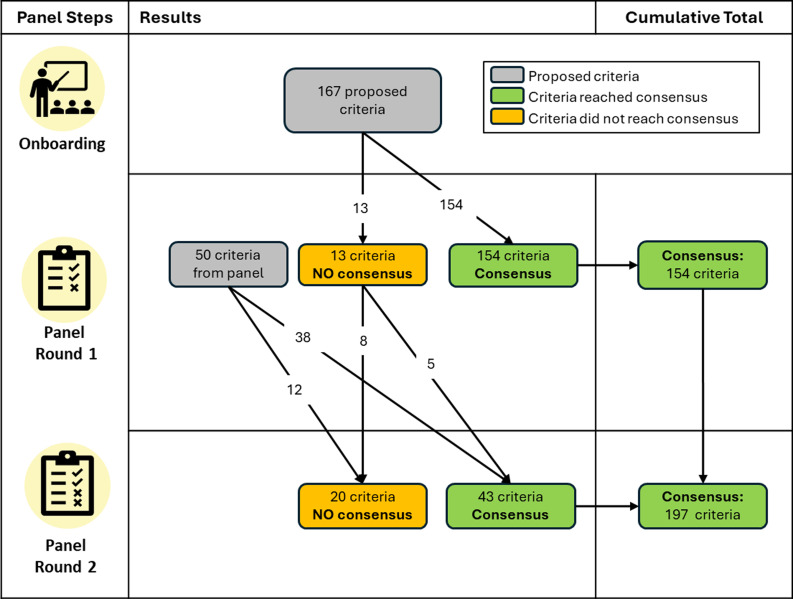
Flowchart of criteria across the Delphi panel rounds.

All 11 experts participated in the Round 1 survey. Of the 167 proposed criteria, panelists reached consensus (at least 70% agreement) that 154 criteria were required (92%; see Figure 1). The proposed criteria on which panelists did not reach consensus fell into three of the eight criteria categories: Who (*N* = 5), Reach (*N* = 4), and How (*N* = 4) (See Supplementary Document 1). These 13 criteria on which panelists failed to reach consensus were discussed during the moderated panel discussion, with 8 of 11 panelists participating.

Within the open text boxes for Round 1, 20 new individual criteria were proposed by panelists, and one new criterion category for “documented evidence,” to be applied across all 30 benefits, was proposed. These were added to the Round 2 Benefits Criteria Survey.

### Benefits criteria survey round 2

**Figure 2.**
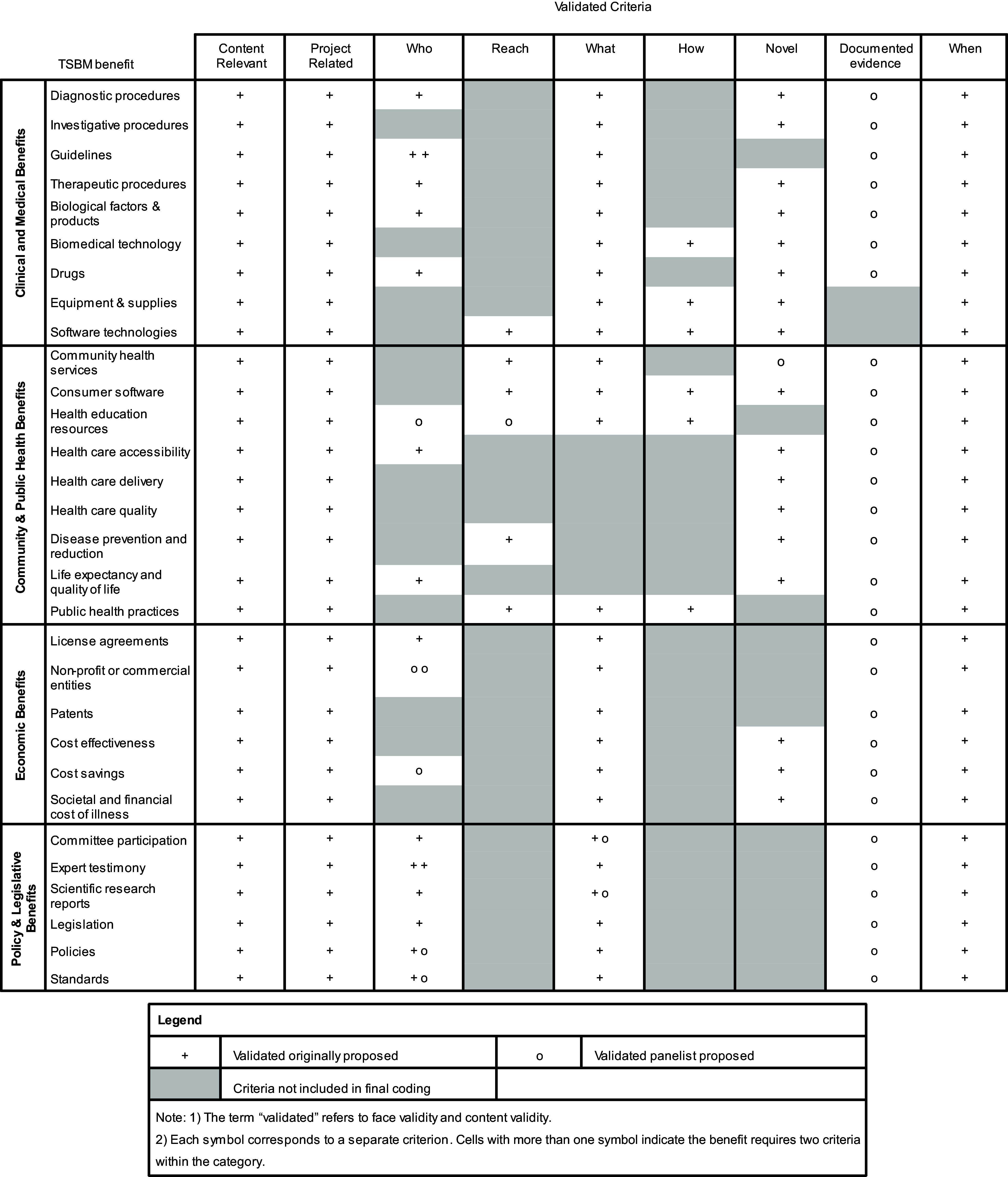
Validated criteria from the Delphi panel.

Nine panelists completed the Round 2 survey. An additional five of the originally proposed criteria reached consensus (> 70% panel agreement), thus increasing the percentage of the originally proposed criteria that were validated (i.e., found to be face and content valid) to 95% (*N* = 159). Consensus was also reached for 10 new panelist-proposed criteria from Round 1, which fell within these categories: Who (*N* = 6), Reach (*N* = 1), What (*N* = 2), and Novel (*N* = 1). The panel also reached consensus that the panelist’s proposed category of “documented evidence” should be applied to 28 of the 30 benefits. In the open text boxes, only 22% of the panel provided specific options for what documented evidence could be provided to verify a given benefit, and nearly half of the options suggested (47.7%) proposed collecting academic publications as evidence of the benefit. Over both rounds, the panel resulted in 197 criteria validated by the panelists, ranging from 5-8 criteria per benefit. Figure 2 illustrates the criteria found to be face and content valid. The panelist-proposed criteria that were not validated can be found in Supplementary Document 2, and Supplementary Document 3 shows wording for the full set of included coding criteria.

## Discussion

The work reported here reflects a response to an identified need for academic research centers to demonstrate impact from funded research projects not only at the individual project level but also at the aggregate research program level. Balancing the complex dynamics of tracking research outcomes and analyzing downstream benefits, we have proposed a set of criteria for analyzing investigator self-reported data on the translational benefits of research using the TSBM framework. A Delphi Panel consisting of experts in evaluation, TSBM data collection, and reporting reached consensus on 197 criteria that fall within 9 categories: Content Relevant, Project Related, Who, Reach, What, How, Novel, Documented Evidence, and When.

Additional work will be required to translate the set of criteria that emerged from the Delphi project into a tool for coding narrative reports of translational science benefits. Strict application of this set of criteria will enable program evaluators to flag instances in which a benefit is claimed as resulting from research, but the qualitative information provided fails to satisfy all of the criteria for that benefit. Options in these cases would include seeking additional information to verify a benefit, rejecting the claimed self-reported benefit, or determining whether satisfaction of some subset of criteria is sufficiently persuasive to count a benefit as having been demonstrated. Given available resources, we recommend seeking additional information from investigators, but also expect that a hierarchy of needed criteria may emerge over time, as evaluators gain more experience applying this list of criteria to a wider range of data.

This Delphi panel considered the unique facets of each individual TSBM benefit and yielded feedback about the criteria that are necessary to verify that each of the reported benefits was the product of a study. The Delphi method in this project included a moderated panel discussion on criteria that did not reach the threshold of consensus in Round 1, which afforded panelists the opportunity to discuss the strengths and/or weaknesses of each criterion and then express their opinion again in the Round 2 survey. This approach mitigated a potential weakness of a traditional Delphi method [27,28], which may lead panelists to decide to adjust responses primarily to reach consensus and would not typically incorporate an opportunity for panelists to discuss the conceptual rationale for their decisions.

The TSBM has been acknowledged as a promising framework for benchmarking the impact of scientific discoveries [29], but most applications of the model generate detailed case studies that require extensive in-depth qualitative and archival investigation (e.g., [30,31]). Considerable resources have been made available by the originators of the model at Washington University to assist program evaluators with the task of constructing case studies [17]. The criteria identified in this study may be useful to support case study efforts by providing a set of criteria that may be consulted when developing cases to narrow in on areas in which there is a need for further information to present a persuasive demonstration of impact.

Furthermore, the potential exists to use the TSBM framework to examine group and programmatic impact of research portfolios. To date, a systematic approach to documenting the translational impacts of a group of projects has not been developed, despite multiple mentions of this potential in the literature [32,33]. The set of criteria identified here offers an initial systematic approach to evaluating qualitative descriptions of benefits and to quantify the translational benefits of a research portfolio.

Having the ability to characterize a group of studies according to their translational benefits is valuable to the research enterprise in several ways. The UCI ICTS has used this methodology to compare the impacts of two groups of studies: one a set of traditional campus-bound research projects, and the other a set of campus-community partnership studies [22]. These findings highlighted that the campus-community partnerships were more likely to result in policy and community-based public health benefits, while the campus-bound projects were more likely to result in clinical and medical benefits. In the absence of a systematized approach, such group comparisons are complex and time-intensive to achieve, yet they are extremely useful to program managers seeking to make decisions about research support and resource allocation.

The results of this Delphi panel study can be used to inform strategies to collect TSBM data reliably and to provide guidance to investigators reporting benefits, such as providing prompts based on these criteria within a survey collecting self-report on the benefits resulting from research. Results will also be used to inform the development of a TSBM coding tool that can be utilized to track research impact and enhance evaluation before and after programmatic innovations. One example of how such an approach might be valuable is in the area of dissemination and implementation research, where it has been suggested that establishing a policy for including community members as co-authors on academic papers might accelerate research dissemination [34]. Tracking translational benefits over time across a research portfolio before and after such a policy is put in place would provide a way to test the impact of the policy.

Additionally, well-rounded evaluation of research impact is enhanced by efforts to triangulate data sources. Evaluators in academic contexts often track bibliometrics as evidence of publication impact, and these approaches would be bolstered by data on direct societal impacts and provide a more comprehensive understanding of the effects of the research. The current approach was built around self-reported benefits, one source out of many data sources that exist on impact. The work described here helps to improve the validity of such self-reported data. In the future, this coding process can also be applied in a systematic way to diverse sources of impact data, such as databases on intellectual property and policy impacts, as well as other publicly available data, to rigorously promote a more robust picture of downstream research impact.

There are reports of research organizations incorporating the TSBM into their tracking and evaluation systems (e.g. [35,36]), and the UCI team’s experience suggests that there are a variety of approaches used to interpret TSBM data and to report the benefits demonstrated from research using qualitative descriptions. Grounded in the UCI team’s in-depth exposure to this process and confirmed by the experts convened in this panel, the set of criteria developed in this study is the first step in creating a TSBM coding process to offer a streamlined and systematic strategy to standardize the translation of qualitative data into aggregate summaries that can be used to characterize the impacts of a complete research portfolio.

## Limitations

Convenience and snowball sampling were used in the present study to identify panelists. The current findings on the criteria to verify TSBM benefits may therefore be specific to the views of the Delphi panel. The panelists are patients, caregivers, researchers, educators, members of their community, and the general public, among other roles that informed their responses. Each panel participant works with a CTSA hub connected to a university, and their perspectives may differ from evaluators in other settings or individuals with other areas of expertise. This commonality may unintentionally limit the usefulness of the coding process to assess research impact beyond academia or limit its broader applicability.

The selection of a subjective level to designate as the panel reaching consensus is a limitation of the Delphi approach, as this level varies widely from study to study [27,28]. This project’s consensus threshold was 70% given the size of the panel, and based on a desire during study planning to include criteria with consensus at or near three-quarters of the panel; a level that is common across studies using this method [28]. A different threshold could have been chosen that altered which criteria reached the inclusion threshold across the Delphi Panel rounds.

The set of criteria yielded by this study is likely to undergo further refinement. The criteria were developed based on the original TSBM framework, which has not been formally updated since 2017. Since then, adaptations to the model have been proposed, with a benefit uniquely created for a recently published case study on the TSBM website [37], a new TSBM domain of Health Equity as an addition to the model [38], as well as indicators with application for implementation science [39]. As the TSBM is revised, criteria will need to be expanded for newly proposed benefits.

## Future directions

Identifying criteria for TSBM coding is a first step towards data standardization in translating qualitative TSBM data into quantitative summaries of research portfolios. Future projects should test the utility and feasibility of TSBM coding across a range of studies to examine its reproducibility and sensitivity to change over time. Work is also needed to explore the feasibility of requiring all criteria identified here in practice and to explore whether a subset of criteria can provide the necessary information to verify that given TSBM benefits have occurred. Some criteria may be difficult or impractical to obtain when applying the coding process. In particular, we recommend further elaboration of the category for “documented evidence” with a need to establish clear guidelines around what constitutes documented evidence for each benefit. Candidates might include published policy documents, press releases, newspaper articles, white papers, collaborating or corroborating research studies that have been published, regulatory approval, governmental bills and laws, institutional records documenting grants or milestones, or review articles (e.g., meta-analyses and scientific reviews). There is a need in the future to establish guidelines for coders and recommendations for identifying strong sources that would constitute “documented evidence” for each of the translational science benefits.

Additional steps to refine the criteria should also seek input from other relevant stakeholder groups, especially those with experience in how activities related to benefits are implemented in applied settings. Relevant stakeholders might include patients, members of the community, clinicians, and individuals making health policies, all of whom would bring unique and valuable insights related to what matters when assessing the likelihood that reported translational science benefits have emerged from given research. Thus, further testing of the criteria that have emerged from this Delphi panel study is needed prior to widespread deployment.

It will also be important to determine what level of training or experience is required for coders to reliably employ the recommended criteria. We plan to test the implementation of the coding criteria across multiple research programs, including assessing interrater reliability, and this will help develop a protocol for the coding process and an associated coding manual. The UCI team also continues to develop digital tools that will streamline coding and is working to create a web-based interface that will make TSBM coding quick and simple for a trained coder to execute. Additional work is needed applying the coding process to research portfolios both at individual sites and across sites. Conducting this analysis on groups of projects can build a body of knowledge to identify trends regarding when and which impacts result from scientific research.

## Supporting information

10.1017/cts.2025.76.sm001 Miovsky et al. supplementary material 1 Miovsky et al. supplementary material

10.1017/cts.2025.76.sm002 Miovsky et al. supplementary material 2 Miovsky et al. supplementary material

10.1017/cts.2025.76.sm003 Miovsky et al. supplementary material 3 Miovsky et al. supplementary material

## Supplementary material

For supplementary material accompanying this paper visit https://doi.org/10.1017/cts.2025.76

## Note

To access the dataset from the Delphi panel visit https://doi.org/10.5061/dryad.zkh1893j9. For questions about the TSBM coding criteria or interest in using the criteria, please contact Nicole Miovsky from UCI ICTS, at nyacono@uci.edu.
