# Supplementary material for: Measuring the aggregated impact of research: Establishing criteria for coding Translational Science Benefits Model data
Source: J Clin Transl Sci. 2025 May 16;9(1):e129. doi: 10.1017/cts.2025.76 (PMC12209970; doi:10.1017/cts.2025.76)
Supplement: Miovsky et al. supplementary material 1 — Miovsky et al. supplementary material [file S2059866125000767sup001.pdf]

Supplementary Document 3

TSBM Coding Criteria Wording

	TSBM Benefit	Content Relevant	Project Related	Who	Reach	What	How	Novel	Documented Evidence	When
Clinical and Medical Benefits	**Diagnostic procedures**	content relevant to the TSBM definition	content indicating the benefit is tied to the project?	who the diagnostic procedure is for?		what the purpose of the diagnostic procedure is for?		how/if it is new or has improved previous diagnostic procedures?	documented evidence of developing the diagnostic procedure?	content indicating the benefit has happened?
	**Investigative procedures**	content relevant to the TSBM definition	content indicating the benefit is tied to the project?			what the purpose of the investigative procedure is?		how/if it is new or has improved previous investigative procedures?	documented evidence of developing the investigative procedure?	content indicating the benefit has happened?
	**Guidelines**	content relevant to the TSBM definition	content indicating the benefit is tied to the project?	the name of the organization issuing the guidelines?<br>who the guidelines are for?		what the content of the guildelines are?			documented evidence of the guidelines?	content indicating the benefit has happened?
	**Therapeutic procedures**	content relevant to the TSBM definition	content indicating the benefit is tied to the project?	who the therapeutic procedure is for?		what the purpose of therapeutic procedure is?		how/if it is new or has improved previous therapeutic procedures?	documented evidence of developing the therapeutic procedure?	content indicating the benefit has happened?
	**Biological factors and products**	content relevant to the TSBM definition	content indicating the benefit is tied to the project?	who the biological factors or products are for?		what the biological factors or products are and their medical purpose?		how/if it is new or has improved previous biological factors or products?	documented evidence of developing the biological factors or products?	content indicating the benefit has happened?
	**Biomedical technology**	content relevant to the TSBM definition	content indicating the benefit is tied to the project?			what the biomedical technology is?	how the biomedical technology is used?	how/if it is new *or* has improved previous biomedical technology?	documented evidence of developing the biomedical technology?	content indicating the benefit has happened?
	**Drugs**	content relevant to the TSBM definition	content indicating the benefit is tied to the project?	who the drug is used by?		what the purpose of the drug is?		how/if it is new or has improved previous drugs?	documented evidence of developing the drug?	content indicating the benefit has happened?
	**Equipment and supplies**	content relevant to the TSBM definition	content indicating the benefit is tied to the project?			what the equipment or supply is?	how the equipment or supply is used?	how/if it is new *or* has improved previous equipment and supplies?		content indicating the benefit has happened?
	**Software technologies**	content relevant to the TSBM definition	content indicating the benefit is tied to the project?		details indicating the software is available to others outside of the project?	what the software technology is?	how the software technology is used?	how/if it is new *or* has improved previous software technologies?		content indicating the benefit has happened?
Community & Public Health Benefits	**Community health services**	content relevant to the TSBM definition	content indicating the benefit is tied to the project?		details indicating the services are provided for individuals in a community? (i.e. instead of provided population wide)	what the community health service is?		how/if it is new *or* has improved community health services?	documented evidence of developing the community health service?	content indicating the benefit has happened?
	**Consumer software**	content relevant to the TSBM definition	content indicating the benefit is tied to the project?		details indicating the software is broadly available to consumers?	what the consumer software is?	how the consumer software is used?	how/if it is new *or* has improved on previous consumer software?	documented evidence of developing the consumer software?	content indicating the benefit has happened?
	**Health education resources**	content relevant to the TSBM definition	content indicating the benefit is tied to the project?	who the users of the health education resources are?	details indicating the health education resource is broadly disseminated?	what the health education resource is?	how the health education resource is used?		documented evidence of developing the health education resource?	content indicating the benefit has happened?
	**Health care accessibility**	content relevant to the TSBM definition	content indicating the benefit is tied to the project?	who health care access has improved for?				how health care access has improved?	documented evidence of improved health care access?	content indicating the benefit has happened?
	**Health care delivery**	content relevant to the TSBM definition	content indicating the benefit is tied to the project?					how health care delivery has improved?	documented evidence of improved health care delivery?	content indicating the benefit has happened?
	**Health care quality**	content relevant to the TSBM definition	content indicating the benefit is tied to the project?					how the quality of care has improved, and standards the quality of care was measured by (if applicable)?	documented evidence of improved quality of care?	content indicating the benefit has happened?
	**Disease prevention and reduction**	content relevant to the TSBM definition	content indicating the benefit is tied to the project?		content indicating disease prevention and reduction is community or population wide?			how disease prevention and reduction has improved?	documented evidence of disease prevention or reduction?	content indicating the benefit has happened?

Note: Current as of March 2025. The included items may be adapted in ongoing projects to assess acceptability and comprehension of the item wording.

Supplementary Document 3

TSBM Coding Criteria Wording

	**Life expectancy and quality of life**	content relevant to the TSBM definition	content indicating the benefit is tied to the project?	who life expectancy and quality of life has improved for?				how life expectancy or quality of life has improved, and what measure of life expectancy or quality of life was used (if applicable)?	documented evidence of improved life expectancy or quality of life?	content indicating the benefit has happened?
	**Public health practices**	content relevant to the TSBM definition	content indicating the benefit is tied to the project?		details indicating the services are provided for whole communities or populations? (instead of care provided for specific individuals)	what the public health service is?	how the public health service was organized or delivered?		documented evidence of organizing or delivering the public health service?	content indicating the benefit has happened?
**Economic Benefits**	**License agreements**	content relevant to the TSBM definition	content indicating the benefit is tied to the project?	who entered into the license agreement?		what the license agreement is for?			documented evidence of the license agreement?	content indicating the benefit has happened?
	**Non-profit or commercial entities**	content relevant to the TSBM definition	content indicating the benefit is tied to the project?	who the population is that receives services from the non-profit or commercial entity?<br>______________<br>the name of the non-profit or commercial entity?		what the non-profit or commercial entity does?			documented evidence of developing the non-profit or commercial entity?	content indicating the benefit has happened?
	**Patents**	content relevant to the TSBM definition	content indicating the benefit is tied to the project?			what the patent was received for?			documented evidence of the patent?	content indicating the benefit has happened?
	**Cost effectiveness**	content relevant to the TSBM definition	content indicating the benefit is tied to the project?			what project activities were done for cost effectiveness?		how cost effectiveness has improved?	documented evidence of improved cost effectiveness?	content indicating the benefit has happened?
	**Cost savings**	content relevant to the TSBM definition	content indicating the benefit is tied to the project?	who experienced cost savings?		what the cost savings are?		how cost savings has improved?	documented evidence of cost savings?	content indicating the benefit has happened?
	**Societal and financial cost of illness**	content relevant to the TSBM definition	content indicating the benefit is tied to the project?			what the reduction in societal and financial costs of illness are?		how societal and financial costs of illness has improved?	documented evidence of reduced societal and financial costs of illness?	content indicating the benefit has happened?
**Policy & Legislative Benefits**	**Committee participation**	content relevant to the TSBM definition	content indicating the benefit is tied to the project?	the name of the committee?		what the committee does?<br>______________<br>what the policy or legislative aims of the committee are?			documented evidence of the committee participation?	content indicating the benefit has happened?
	**Expert testimony**	content relevant to the TSBM definition	content indicating the benefit is tied to the project?	who gave the expert testimony?<br>______________<br>the name of the entity the testimony was given to?		what content was presented in the expert testimony?			documented evidence of the expert testimony?	content indicating the benefit has happened?
	**Scientific research reports**	content relevant to the TSBM definition	content indicating the benefit is tied to the project?	who the target audience of the document is?		what the purpose and topic of the document is?<br>______________<br>what the name of the report is?			documented evidence of developing the report?	content indicating the benefit has happened?
	**Legislation**	content relevant to the TSBM definition	content indicating the benefit is tied to the project?	who the legislative body is that passed it or the name of the legislation?		what the content of the legislation is?			documented evidence of the legislation?	content indicating the benefit has happened?
	**Policies**	content relevant to the TSBM definition	content indicating the benefit is tied to the project?	who the entity is that created the policy?<br>______________<br>who the population is that the policy change benefits?		what the content of the policy is?			documented evidence of the policies?	content indicating the benefit has happened?
	**Standards**	content relevant to the TSBM definition	content indicating the benefit is tied to the project?	a description of the entity that issued the standards?<br>______________<br>who the population is that the standards benefit?		what the content of the standards are?			documented evidence of the standards?	content indicating the benefit has happened?

Note: Current as of March 2025. The included items may be adapted in ongoing projects to assess acceptability and comprehension of the item wording.
