# Supplementary material for: Measuring the aggregated impact of research: Establishing criteria for coding Translational Science Benefits Model data
Source: J Clin Transl Sci. 2025 May 16;9(1):e129. doi: 10.1017/cts.2025.76 (PMC12209970; doi:10.1017/cts.2025.76)
Supplement: Miovsky et al. supplementary material 2 — Miovsky et al. supplementary material [file S2059866125000767sup002.pdf]

Supplementary Document 1

Table of Originally Proposed Criteria that did not Reach the Consensus Threshold in Round 1 and Percent Agreement during Round 2.

Criteria Category	TSBM Benefit	% Agree	
		**Round 1**	**Round 2**
**Who**	Diagnostic procedures	54.5%	**>70%**
	Therapeutic procedures	63.6%	**>70%**
	Biological factors and products	54.5%	**>70%**
	Drugs	54.5%	**>70%**
	Committee participation	63.6%	44.4%
**Reach**	Guidelines	45.5%	33.3%
	Software technologies	63.6%	**>70%**
	Health care delivery	54.5%	22.2%
	Standards	36.4%	22.2%
**How**	Diagnostic procedures	45.5%	33.3%
	Investigative procedures	45.5%	33.3%
	Therapeutic procedures	36.4%	33.3%
	Committee participation	54.5%	66.7%

>70% = reached consensus
