# Supplementary material for: Measuring the aggregated impact of research: Establishing criteria for coding Translational Science Benefits Model data
Source: J Clin Transl Sci. 2025 May 16;9(1):e129. doi: 10.1017/cts.2025.76 (PMC12209970; doi:10.1017/cts.2025.76)
Supplement: Miovsky et al. supplementary material 3 — Miovsky et al. supplementary material [file S2059866125000767sup003.pdf]

Supplementary Document 2

Table of Percent Agreement for Criteria Proposed by Panelists in Round 1 that did not Reach Consensus in Round 2.

	Translational Science Benefit	Specific Criterion	% Agree Round 2
**Panelist proposed criteria not reaching consensus**	Therapeutic procedures	The patient-reported value of therapy	33.3%
	Equipment & supplies	Broad access to or use of the equipment or supply	33.3%
	Equipment & supplies	Documented evidence of developing the equipment or supply	66.7%
	Software technologies	Documented evidence of developing the software technology	66.7%
	Community health services	Broad access to or use of the community health services	44.4%
	Consumer software	The value of the software to the consumer	66.7%
	Health care accessibility	How broad the increase in health care access is	55.6%
	Health care quality	How broad the improvement in health care quality is	55.6%
	Public health practices	How use of the public health practice is being spread	33.3%
	Societal and financial cost of illness	Who experiences reduced societal and financial costs of illness	66.7%
	Scientific research reports	How broadly disseminated or available the report is	44.4%
	Policies	How the policy is used to guide decision-making	33.3%
